# Associations of community knowledge, perceptions, and practices related to zoonotic disease with sociodemographic factors in and around Chiro Town, Eastern Ethiopia: a cross-sectional study

**DOI:** 10.1186/s42522-024-00105-9

**Published:** 2024-06-07

**Authors:** Abdulaziz Abrahim, Bantayehu Bekele, Muhidin Tahir, Sali Ahmed, Lencho Ahmedin

**Affiliations:** 1 Department of Biology, College of Natural and Computational Science, Oda Bultum University, P.O. BOX 226, Chiro, Ethiopia; 2 Department of Nursing, School of Nursing and Mid-Wife, College of Medicine and Health Sciences, Haramaya University, P.O. Box 138, Haramaya, Ethiopia

**Keywords:** Anthrax, Bovine tuberculosis, Brucellosis, Knowledge, Rabies

## Abstract

**Background:**

Zoonoses are infectious diseases that are transmitted from animals to humans. Studying the knowledge, perceptions and practices of communities related to zoonoses and the associated risk factors is crucial for effective control and prevention. This study aimed to assess the knowledge, perceptions, and practices of respondents on zoonoses and the associated risk factors in and around Chiro town, Ethiopia. Zoonotic diseases, such as rabies, anthrax, bovine tuberculosis, and brucellosis, pose a direct threat to health and livelihoods in the communities where they occur. These diseases emerge due to a combination of human-animal interactions, migration, and contact with wildlife and their respective parasites and vectors. Hence, recognizing residents’ perceptions, knowledge, and practices is crucial for effectively minimizing risks.

**Methods:**

A cross-sectional study was conducted from January 2020 to July 2021 in and around Chiro town using a pretested close-ended questionnaire. A total of 350 respondents were selected using simple random sampling methods. The questionnaire included information on the sociodemographic status of the respondents and questions concerning the knowledge, perceptions, and practices of the participants regarding the selected zoonotic diseases. The associations of knowledge, perceptions, and practices related to zoonoses with zoonotic risk factors were analysed using chi-square tests.

**Results:**

The study revealed that 82.9% of the respondents had knowledge of bovine tuberculosis, followed by knowledge of rabies (80%), knowledge of anthrax (45.1%), and knowledge of brucellosis (24.3%). Males had greater knowledge of bovine tuberculosis (84.8%), followed by rabies (79.8%) and anthrax (48.6%), while females had greater knowledge of brucellosis (23.6%). The most cited source of information was radio (68%). Most respondents mentioned the outbreaks of rabies *(*62.5%), bovine tuberculosis (53.2%), anthrax (35.6%), and brucellosis (15.7%). Respondents with higher educational levels and urban residents had more knowledge of zoonoses. More than 75% of respondents had a good perception of the transmission of zoonotic disease from animals, and the practice of consuming raw milk or raw/undercooked meat and sharing the same house with animals was high.

**Conclusion:**

The majority of respondents reported that they had knowledge of bovine tuberculosis and rabies, but lower knowledge and perceptions were reported for anthrax and brucellosis. These findings illustrate the need for collaboration among animal, human and environmental health offices in one health approach to prevent and control zoonotic disease.

**Supplementary Information:**

The online version contains supplementary material available at 10.1186/s42522-024-00105-9.

## Introduction

Zoonoses are infectious diseases that are transmitted between animals and humans by means of numerous groups of viruses, bacteria, fungi, Rickettsia, prions, and parasites in a variety of animal reservoirs, including wildlife, livestock, pet animals, and birds [1]. Among the prevalent zoonotic diseases in developing countries are anthrax, bovine tuberculosis, brucellosis, rabies, cysticercosis, echinococcosis, Japanese encephalitis, leptospirosis, and trypanosomiasis. Risk factors for zoonotic diseases include activities such as animal slaughter, handling and preparing food of animal origin, consuming raw or undercooked animal products, demographic factors and inadequate public health measures [2, 3]. Zoonoses contribute significantly to the emergence and re-emergence of infections in low- and middle-income countries worldwide, including Ethiopia [4].

The top five prioritized zoonotic diseases in Ethiopia are rabies, anthrax, brucellosis, Rift Valley fever, and highly pathogenic avian influenza [5], 32]. Rabies is a particularly deadly disease caused by the *rabies virus* and is transmitted to humans through the bite of rabid dogs or sometimes from domestic dogs to wildlife, such as the endangered Ethiopian wolf. Anthrax is another common zoonotic disease caused by *Bacillus anthracis* that primarily affects animals such as cattle, goats, and sheep. Humans can contract anthrax by consuming raw or undercooked meat from infected animals or through close contact with them [5]. Bovine brucellosis, primarily caused by *Brucella abortus* but occasionally by *Brucella melitensis* and *Brucella suis*, poses a significant challenge to cattle production and public health in Ethiopia. Despite being the second most dangerous zoonotic disease globally after rabies, brucellosis is often neglected in Ethiopia, although it has high public health and economic impacts [6]. The disease can be transmitted to humans through the ingestion of raw milk or meat, direct contact with infected animal uterine discharge, or inhalation of airborne bacteria [7]. Bovine tuberculosis (TB) is a major cause of infection and mortality worldwide and caused by *Mycobacterium bovis* in cattle [8]. In Ethiopia, reports indicate that the prevalence of bovine TB can reach up to 50% in intensive dairy production systems. The disease can spread through the consumption of raw milk or undercooked meat and close contact with infected animals [9, 10].

Over the past decade, zoonotic diseases have caused significant economic losses globally, with direct losses of $20 billion and indirect losses of $200 billion [10]. With 50 million people worldwide impacted, 80% of developing countries are affected the most, resulting in 2.2 million deaths annually. Annually, there are 10,000–100,000 human anthrax cases worldwide, with a substantial number reported in developing countries such as Ethiopia, Chad, Zimbabwe, Zambia, and India [11]. Although more than 80% of the population in Ethiopia relies on agriculture, with approximately 65% depending on livestock production, the country ranks high in terms of the health burden of zoonotic diseases. Among the more prevalent zoonoses in Ethiopia, anthrax and rabies are the most common zoonotic diseases [11]. The country has one of the highest rabies infection rates of approximately 10,000 anthrax cases annually [12]. Additionally, a high seroprevalence of bovine and human brucellosis has been reported in various parts of Ethiopia, leading to significant economic losses in animals and health issues in humans [10]. Recent studies have shown very high rates of brucellosis in different regions of Ethiopia, including the Afar (48.3%), Somali (34.9%), Oromia (34.1%), Southern Nation Nationality People of Ethiopia (SNNP) (29.5%), Amhara (5.3%), Addis Ababa (4.8%), and Sidama (3.78%) regions [13]. In addition, a higher prevalence of bovine tuberculosis was reported all over the country in cattle managed under intensive management systems, with a higher prevalence in extensive systems [14].

The main constraints of livestock production in Ethiopia include zoonotic diseases such as brucellosis, rabies, Cysticercus bovis, bovine tuberculosis, and anthrax, along with nutritional shortages, traditional husbandry practices, water scarcity, and poor marketing systems [15]. Cultural influences such as consuming raw meat and living closely with animals exacerbate the transmission of zoonotic diseases. Challenges such as limited diagnostic capacity, lack of awareness about zoonotic diseases among communities, poor integration between the animal and human health sectors, and insufficient One Health awareness contribute to the spread of zoonotic diseases [16]. Overall, the abovementioned problems are coupled with close contact between humans and animals, and a large population of low-income livestock farmers increases the risk of zoonotic disease and contributes to the transmission of these diseases [11]. This problem is prevalent in rural communities of Ethiopia, where animals and humans share the same rooms [17] and where there is a widespread habit of consuming raw meat in the form of minced meat (“kitfo”) and steak (“Kurt”) [18]. The practice of consuming raw milk and meat together with handling sick animals and animal products with bare hands accelerates the spread of zoonotic diseases such as anthrax and bovine tuberculosis [11], toxoplasmosis and brucellosis [14], and hydatidosis. For example, according to reports from the Ethiopian Central Statistical Agency, less than 1% of milk is consumed in the form of pasteurized milk [19].

Studies have shown that the success of reducing the public health significance of zoonotic diseases greatly depends on the level of cooperation between the medical and veterinary sectors in the diagnosis of zoonosis, exchange of information, organization of shared surveillance systems, providing common training of staff, and creation of community awareness by providing education and training to communities are considerably decreases the prevalence of livelihood-related diseases. Improving the knowledge of consumers concerning the risks related to zoonotic diseases linked to the consumption of animal products and the adoption of protective measures is essential for effective control and prevention policies [14].

However, inadequate information exists on public risk perceptions and protective measures for zoonotic diseases in Ethiopia, mainly in the study area [20, and 14]. This limited information may be related to sociocultural practices, illiteracy, the number of families, and income [18]. Therefore, it is essential to provide capacity-building training for health professionals and conduct community awareness campaigns through health extension workers, public health education on the risks of consuming food of animal origin and adopting a One Health approach, which unites medical, veterinary, and environmental expertise and will help governments, businesses organization, and civil society realize persistent health for people, animals, and environments [14, 21, 22]. The aim of this study was to assess the knowledge, perceptions, and practices of the community regarding selected zoonoses (rabies, anthrax, brucellosis, and bovine tuberculosis) and the associations of different demographic risk factors with the knowledge, perceptions, and practices of the community in and around Chiro town, Western Hararghe Zone, Eastern Ethiopia. These findings can be used by concerned government structures, including federal, Zonal, and town human health sectors; livestock sectors; nongovernmental organizations; and various stakeholders to take proper measures to prevent and control zoonosis.

## Methods

### Description of the study area

**Fig. 1:**
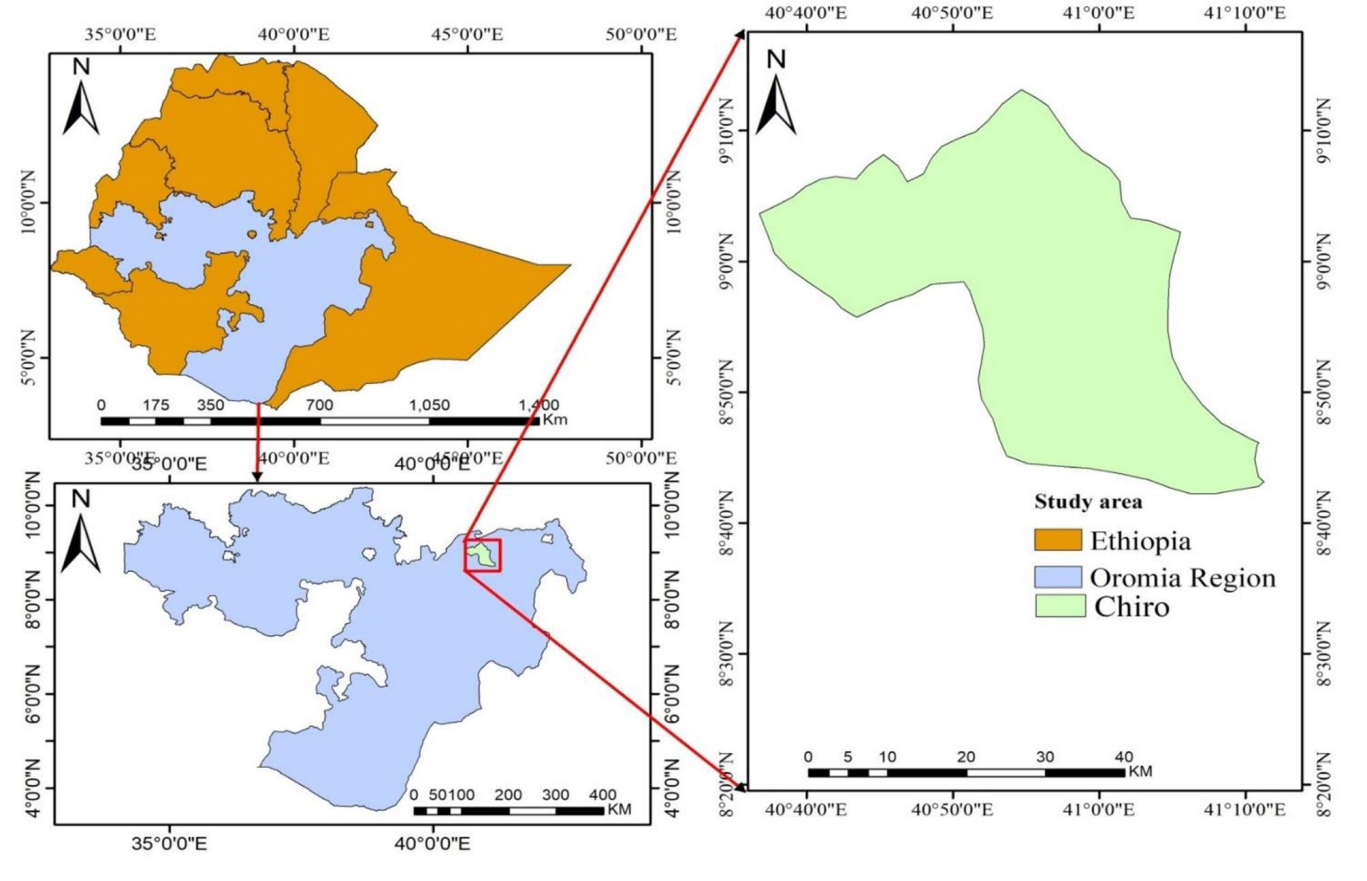
Map of the study area

This cross-sectional study was conducted from January 2020 to July 2021 in and around Chiro town, west Hararghe zone, eastern Ethiopia (Fig. 1). Chiro town is bounded by the Shebelle River to the south, Arsi to the southwest, the Somali Region to the north, and the east Hararghe to the east West Hararghe Zone and has a total population of 1,871,706, an increase of 47.16% over the 2007 census, of whom 958,861 were male and 912,845 were female, with an area of 15,065.86 square kilometres and a population density of 124.23/km^2^ [23]. Chiro town has a total population of 33,643 people, of whom 18,092 are male and 15,551 are female, and the estimated population density of Chiro town in 2022 will be 334.7/km2, with an annual growth rate of 2.2% from 2007 to 2022 [23].

### Study design, population, and determination of sample size

A cross-sectional study was carried out from January 2020 to July 2021 by using close-ended questionnaires and interviews. The study population included residents in and around Chiro town who were grouped into four groups, including household farmers, teachers, students, and other residents, by a stratified random sampling technique, and the respondents were selected by using a simple random sampling method from each group. Thus, the required sample size for this study was estimated by considering the formula given by Yemane [24] for the questionnaire survey. A 95% confidence interval was used to calculate the sample size. A total of 350 respondents were selected to increase precision.

n = N/1 + N (e) ^2^_,_ where n is the total sample, N is the total population, and e is the level of precision (0.05).

The sample size of each stratum was determined by the following formula: nh = (Nh/N) * n

where nh is the sample size for stratum h, Nh is the population size for stratum h, N is the total population size, and n is the total sample size.

### Data collection

A total of 350 respondents were stratified into farmers (200), students (85), teachers (23), and other residents (42) by considering them as representatives of the study population. During data collection the English version of the questionnaire was first prepared and then translated into the local language (Afaan Oromo) and back-translated into English by a third person to verify uniformity. The collected questionnaires included 12 questions for assessing knowledge, 7 questions for assessing the general perceptions of respondents and transmission route, and 20 questions on the practices of respondents regarding zoonosis (S1, File). The data were collected after training was delivered to the data collectors, and the data collection tools (questionnaire) were pretested for their clarity, sequence, applicability, and validity. Then, the questionnaire was modified, and the second version was used to collect the data. Each day, the completeness and consistency of the questionnaires were checked to ensure the quality of the collected data. Finally, the data were cross-checked using double data entry. The questionnaire was valid and reliable because it was adapted from the World Health Organization and similar studies [1]. The questionnaire included questions that can evaluate the sociodemographic factors of the respondents, the respondents’ general knowledge of zoonotic diseases and transmission, and the respondents’ knowledge of zoonotic diseases such as rabies, anthrax, brucellosis, and bovine tuberculosis. This study also compared the knowledge, attitudes and practices of respondents towards zoonoses with socio-demographic factors.

### Ethics approval and consent to participate

Permission for this cross-sectional study was obtained from the Ethical Review Committee of Oda Bultum University, and verbal permission from each study participant was obtained after a clear explanation of the purpose and benefit of the study was given. The study participants were informed that participation was entirely voluntary and that their decision to participate did not affect them or any of his/her household members. They were also informed that they had the right to refuse to answer any of the questions that made them feel uncomfortable. Codes were given and used for each participant instead of their names to maintain the confidentiality of the responses given by them.

### Data analysis

The data collected through the questionnaire were properly coded and analysed using SPSS software (version 26). Descriptive statistics such as frequency/percentages were used to compute the knowledge, perceptions and practices of respondents regarding zoonotic disease. The association between the awareness of respondents and different sociodemographic factors was analysed by using the Pearson chi-square test, and the *p* value was considered to represent a significant difference at the 5% level.

## Results

### Sociodemographic status of the respondents

Overall, 350 respondents participated in the study, for a response rate of 100%. The sociodemographic factors of the respondents, i.e., age group, education level, occupation, sex, and income, were included in the study to evaluate whether the mentioned factors were associated with the knowledge, perceptions, and practices of the community regarding selected zoonotic diseases (rabies, anthrax, brucellosis, and bovine tuberculosis). Out of the total population, 350 respondents were grouped by a stratified random sampling method into farmers (200), students (85), teachers (23), and other residents (42) to assess their knowledge (if they knew the specific selected disease). Of the 350 total respondents who participated in the study, 70% were males, 30% were females, and 37.7% were illiterate, 62.6% were rural residents, and 37.4% were urban residents. The majority of the respondents were farmers (57.1%), followed by students (24.3%), other administrative workers (12%) and teachers (6.6%). Some (62.9%) of the respondents were single, 31.4% were married, and 5.7% were divorced. Close to half of the respondents (48.6%) obtained 500–999 birr/$8.33-$16.65 monthly income, followed by 1000–3000 birr/$16.67-$50 (28%), < 500/$8.33 (8.6%) and 9000/$150 and above (5.7%) (Table 1).

**Table 1:** Sociodemographic status of the respondents (*n* = 350)

Variables Category		Frequency (%)
Sex	Male	245(70%)
	Female	110(30%)
Residence	Urban	131(37.4%)
	Rural	219(62.6%)
Educational Status	Illiterate	132(37.7%)
	Elementary	83(23.7%)
	High school	60(17.1%)
	Preparatory	50(14.3%)
	Above diploma	25(7.2%)
Occupation	Teacher	23(6.6%)
	Student	85(24.3%)
	Farmer	200(57.1%)
	Other	42(12%)
Marital status	Single	220(62.9%)
	Married	110(31.4%)
	Divorced	20(5.7%)
Monthly income (in Birr/US dollars)	Below 500 birr/$8.33	30(8.6%)
	500–999 birr/$8.33-$16.65	170(48.6%)
	1000–3000/$16.67- $50	98(28%)
	3001–5000/$ 50.02-$83.33	22(6.3%)
	5001–8000/$ 83.35-$133.33	10(2.8%)
	9000/$150 and above	20(5.7%)

### Respondents’ knowledge of zoonotic disease

**Fig. 2:**
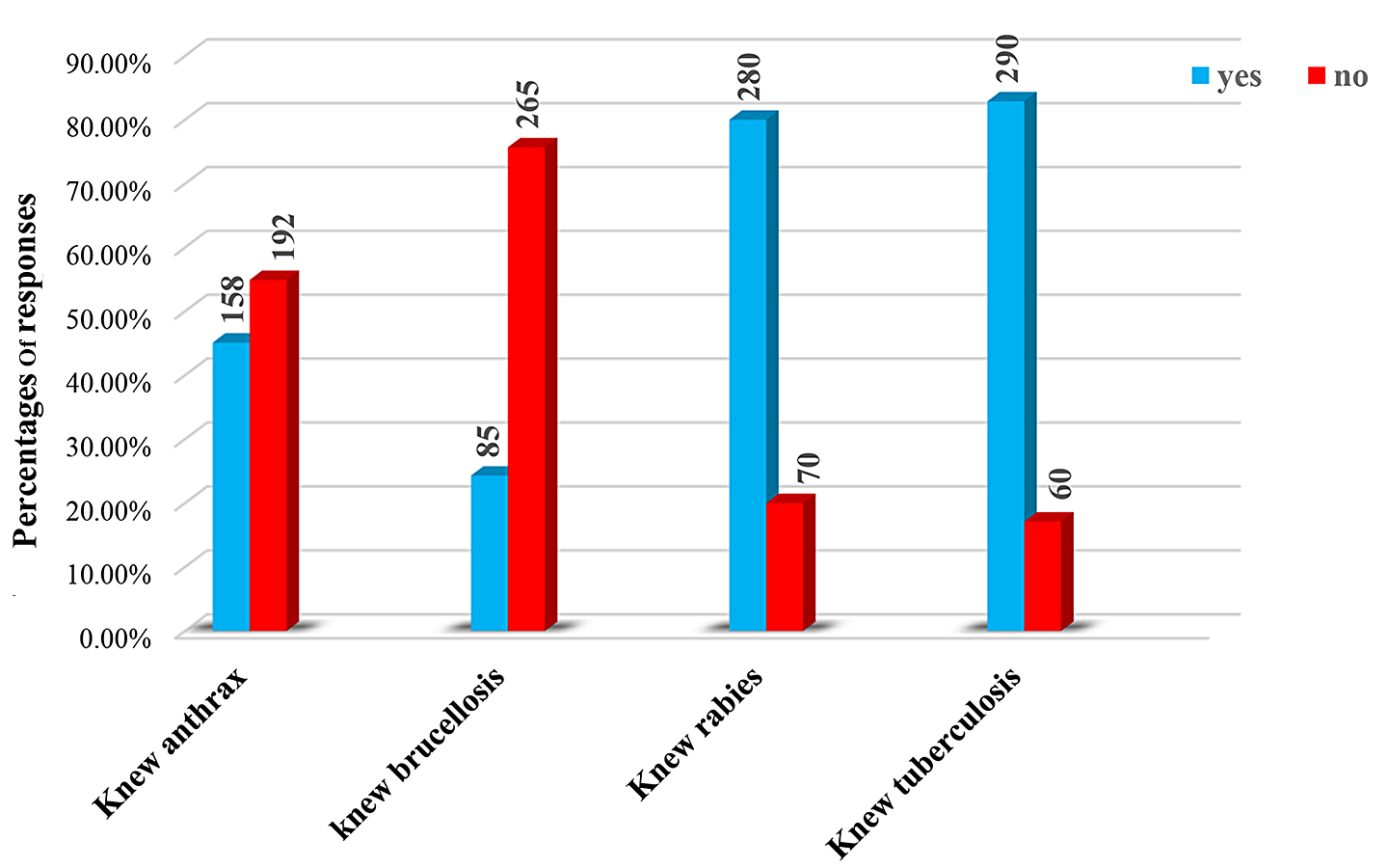
Knowledge of community regarding zoonotic disease

The study revealed that 82.9%, 80%, 45.1%, and 24.3% of respondents had knowledge of tuberculosis, rabies, anthrax, and brucellosis, respectively (Fig. 2).

### Sources of information for respondents

**Fig. 3:**
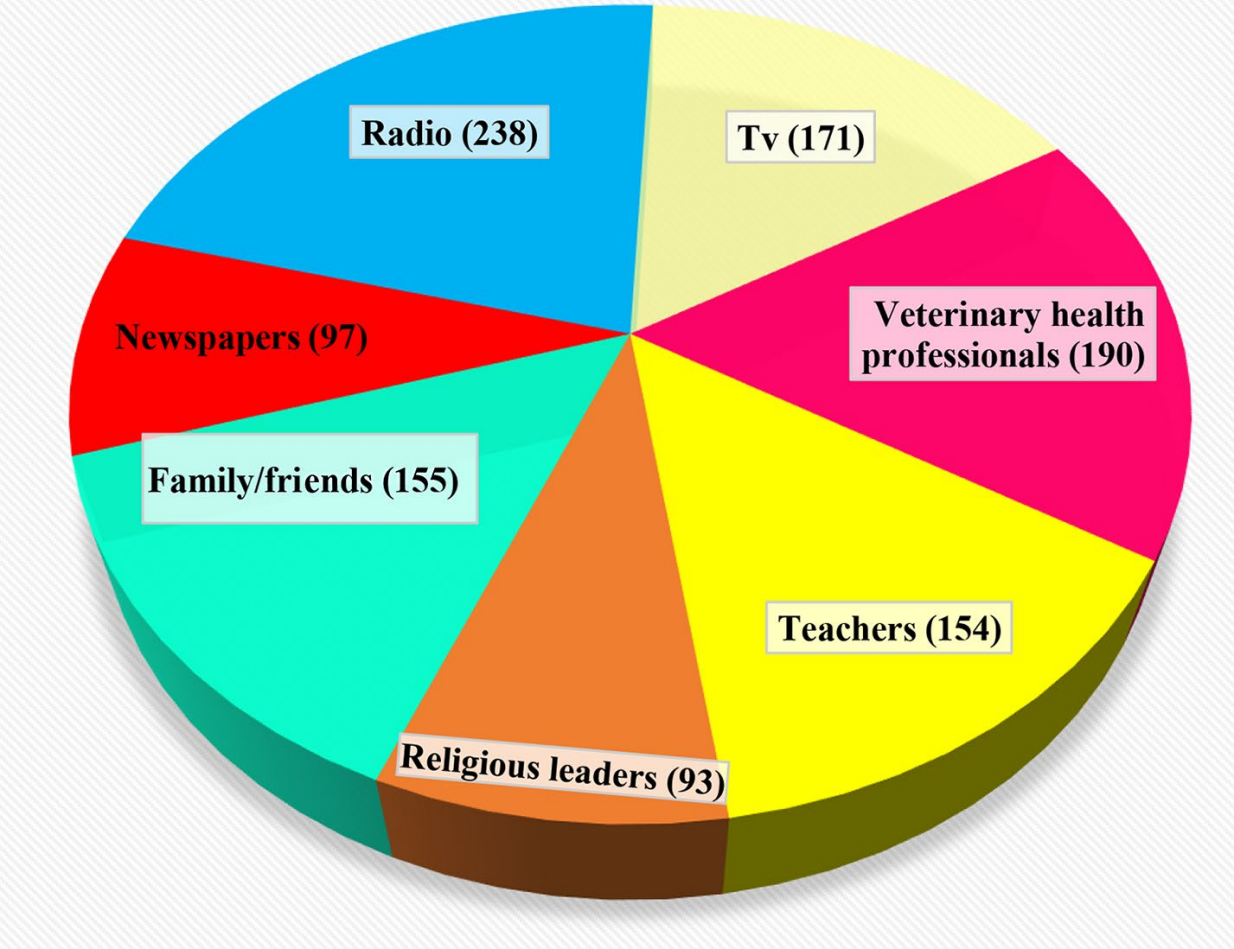
Source of information of respondents

The sources of information for the respondents were radio 238 (68%), TV 171 (48.9%), family and friends 155 (48%), teachers 154 (44.3%), veterinary health professionals 190 (44%), newspapers 97 (27.4%), and religious leaders 93 (15%) (Fig. 3).

### Zoonotic disease knowledge among different sociodemographic factors

Below Table 2. Shown that the associations between respondents’ knowledge and sex, age, residence, educational status and occupation. The study revealed that of 240 male and 110 female respondents, 84.8%, 79.8%, and 48.6% of males had knowledge of bovine tuberculosis, rabies, and anthrax, respectively, while 23.5% of females had knowledge of zoonotic brucellosis. Thus, the associations between the sex of the respondents and knowledge of rabies, brucellosis, and tuberculosis were not statistically significant (*p* > 0.05), but a significant association was found for anthrax (*p* = 0.05). In terms of educational level, respondents with a high school education (*n* = 60), a preparatory education (*n* = 50), or above (*n* = 25) had more knowledge of the mentioned zoonotic disease than did those with a primary education (*n* = 83) or who were illiterate (*n* = 132) (Table 2). Significant associations were found for anthrax (*p* = 0.05), rabies (*p* = 0.05), and tuberculosis (*p* = 0.05), but not for brucellosis (*p* > 0.05). This finding also showed that among the 131 urban and 219 rural respondents included in this study, 89.3%, 83.2%, 50.4%, and (22.1%) of the urban respondents had knowledge of bovine tuberculosis, rabies, anthrax and brucellosis, respectively, while rural residents had higher knowledge of bovine tuberculosis (78.5%), rabies (77.2%), anthrax (41.5%), and brucellosis (24.2%). Thus, the associations between the respondents’ residence and knowledge of zoonotic anthrax, rabies, and brucellosis were not significantly different (*p* > 0.05), but the significant association was found for zoonotic tuberculosis (*p* = 0.05). Regarding the occupations of the respondents, teachers (23), students (85), farmers (200) and others (42), including administrative workers, merchants, health professionals, etc., participated in this cross-sectional study, and among these 96.5%, 89.4%, and 70.6% of the students had knowledge of rabies, bovine tuberculosis, and anthrax, respectively, while farmers had greater knowledge of brucellosis (25.5%). The association was found to be significant for anthrax and bovine tuberculosis (*p* = 0.05), but it was not significant for rabies or brucellosis (*p* > 0.05).

**Table 2:** Comparison of respondents’ knowledge of zoonotic disease with sociodemographic factors (*n* = 350)

	Category	Response	Do you know the following zoonotic disease?											
			Anthrax F (%)	X^2^	*P*- value	Rabies F (%)	X^2^	*P*- value	Brucellosis F (%)	X^2^	*P*- value	Tuberculosis F (%)	X^2^	*P*- value
Sex	Male (*n* = 240)	Yes	118(48.2%)	3.737	0.050*	194(79. 2%)	0. 458		55(22.4%)	0.029	0.864	207(84.5%)	3.439	0. 064
	Female (*n* = 110)	Yes	40(36.4%)			83(75.4%)			26(23.6%)			81(73.6%)		
Residence	Urban (*n* = 131)	Yes	66(50.4%)	2.509	0. 285	109(83.2%)	1.199	0.549	29(22.1%)	0.693	0. 707	117(89.3%)	8.254	0. 016*
	Rural (*n* = 219)	Yes	91(41.5%)			169(77.2%)			53(24.2%)			172(78.5%)		
Education level	Illiterate (*n* = 132)	Yes	54(40.9%)	39.272	0. 000*	99(75%)	10.430	0. 034*	31(23.5%)	1.455	0.835	105(79.5%)	11.159	0.025*
	Elementary (*n* = 83)	Yes	26(31.3%)			69(83.1%)			20(24.1%)			63(75.9%)		
	High school (*n* = 60)	Yes	20(33.3%)			40(66.7%)			11(18.3%)			48(80%)		
	Preparatory (*n* = 50)	Yes	44(88%)			47(94%)			12(24%)			42(84%)		
	diploma and above (*n* = 25)	Yes	12(48%)			21(84%)			7(28%)			20(80%)		
Occupation	Teacher (*n* = 23)	Yes	5(21.7%)	14.932	0. 002*	9(39.1%)	3.031	0.387	2(8.7%)	1.267	0.737	9(39.1%)	24.831	0.000*
	Student (*n* = 85)	Yes	60(70.6%)			82(96.5%)			23(27.1%)			81(89.4%)		
	Farmer (*n* = 200)	Yes	77(38.5%)			154(77%)			51(25.5%)			149(74.5%)		
	Other (*n* = 42)	Yes	16(38.1%)			33(78.6%)			7(16.7%)			33 (78.6%)		

Note that * indicates a significant difference at a *p* = 0.05, **X**^**2**^ = Chi-square

### Perception of respondents on the transmission of zoonosis with different educational levels

The results of the current study regarding the perceptions of respondents on the transmission of zoonosis across educational levels indicated that more than half of the respondents had good perceptions of the transmission of zoonotic disease from animals to humans (Table 3). A total of 92%, 86.7%, 80%, 77.3%, and 52% of the respondents with the educational levels of preparatory, primary school, high school, illiterate and diploma and above, respectively, perceived zoonotic disease transmission from animals to humans. The association between respondents’ perceptions of the transmission of zoonosis and education level was found to be significant (*p* = 0.05). Nearly all respondents agreed on the transmission of zoonosis through drinking contaminated water and raw milk, eating under cooked or raw meat, and sharing the same house with domestic animals, and a lower perception was observed regarding transmission through handling animals with cuts/wounds across each education level, with more perception was reported by illiterate respondents (55.3%) and respondents who had a primary education (54.2%) than respondents with the educational levels of secondary (41.7%), preparatory (40%), and diploma and above (17.4%) (Table 3). The associations of perceptions of respondents regarding the transmission of zoonosis through drinking contaminated water, sharing the same house with domestic animals, and handling cuts/wounds with bare hands with education level were statistically significant (*p* = 0.05), but for drinking raw milk and eating undercooked/raw meat, the associations were not significant (*p* > 0.05). Preparatory students (100%) had a high awareness of the transmission of rabies by biting infected dogs, and the association of the transmission of rabies by infected dog bites across educational levels was not significant (*p* > 0.05).

**Table 3:** Perception of respondents on the transmission of zoonotic disease with different education levels (*n* = 350)

Variable	Responses	Perception on transmission of zoonosis Vs educational level						
		Illiterate F (%), *n* = 132	Primary F (%), *n* = 83	Secondary F (%), *n* = 60	Preparatory F (%), *n* = 50	Diploma and above F (%), *n* = 25	X^2^	*P*- value
Transmission from animal to humans	Yes	102(77.3%)	72(86.7%)	48(80.0%)	46(92%)	13(52%)	21.122	0.000*
If yes how?	Drinking contaminated water	76(57.6%)	72(86.7%)	53(88.3%)	49(98%)	22(95.7%)	50.236	0.176
	Drinking raw milk	119(90.1%)	77(92.8%)	55(91.7%)	43(86%)	23(100.0%)	11.475	0.176
	Eating raw or undercooked meat	119(90.1%)	73(87.9%)	48(80%)	45(90%)	20(87.0%)	15.207	0.636
	Handling with cuts/wounds animals	73(55.3%)	45(54.2%)	25(41.7%)	20(40%)	4(17.4%)	2.546	0.004*
	Sharing the same room with animals	65(49.2%)	67(80.7%)	44(73.3%)	42(84%)	22(95.7%)	40.225	0.000*
Biting of infected dogs can contract rabies to humans?	Yes	121(91.7%)	73(87.9%)	53(86.9%)	50(100%)	21(84%)	1.416	0.84

Note that * indicates a significant difference at a *p* = 0.05, **X**^**2**^ = Chi-square

### Comparison of the practices of respondents toward zoonosis with educational education levels

A comparison of the practices of respondents across educational levels indicated that more than 80% of respondents reported receiving a rabies vaccination after rabid dog bite and most of the respondents also mentioned receiving anthrax vaccination once a year, while a lower percentage of respondents reported receiving anthrax vaccination twice, never available or always available (Table 4**)**. The association was not statistically significant for both vaccination after rabid dog bite and availability of vaccine against anthrax (*p* > 0.05). The literate informants always washed their hands with soap after touching infected animals, while a low number of respondents washed their hands with soap only before eating food, and this association was not statistically significant (*p* > 0.05). Approximately 52.3%, 69.5%, 58.6%, 40.7%, and 39.1% of illiterate individuals and those with primary, secondary, preparatory, and diploma and above education, respectively, regularly vaccinating their dogs. The association between dog vaccination and education level was statistically significant (*p* = 0.05). A large number of respondents with different educational levels reported washing their hands with soap after a rabid dog bite, followed by healing with hot paper and applying chili powder (*p* = 0.05) (Table 4). Regardless of educational level, most respondents reported taking an infected person to the nearest health center. The association between action taken for rabid dog bites and practices for infected individuals across educational levels was statistically significant (*p* = 0.05).

**Table 4:** Comparison of the practice of participants with their educational levels (*n* = 350)

Variable	Responses	Practice Vs educational levels						
		Illiterate F (%), *n* = 132	Primary F (%), *n* = 83	Secondary F (%), *n* = 60	Preparatory F (%) *n* = 50	Diploma and above F (%) *n* = 25	X^2^	*P* value
Vaccination after rabid dog bite	Yes	110(82.1%)	73(86.9%)	46(76.7%)	43(81.1%)	21(91.3%)	3.892	0.412
How often vaccination against anthrax given?	Once a year	81(60.4%)	35(41.7%)	32(54.2%)	35(64.8%)	14(63.6%)		
	Twice a year	24(17.9%)	20(24.1%)	12(20.3%)	11(20.4%)	5(22.7%)		
	Never available	26(19.4%)	7(8.3%)	3(5.0%)	1(1.9%)	5(21.7%)		
	always available	19(14.2%)	25(29.8%)	12(20.0%)	5(9.3%)	0(0.0%)		
washing your hands with soap after touching animals	Yes I do it always	100(74.6%)	57(68.7%)	46(76.6%)	43(79.7%)	19(82.6%)	12.064	0.740
	No i don’t	10(7.5%)	6(7.2%)	5(8.3%)	4(7.4%)	1(4.3%)		
	Some times	16(11.9%)	15(18.1%)	6(10.0%)	5(9.3%)	2(8.7%)		
	when i eat food	8(6.0%)	5(6.0%)	3(5.0%)	2(3.7%)	1(4.3%)		
Vaccinate your dog regularly?	Yes	69(52.3%)	57(69.5%)	34(58.6%)	22(40.7%)	9(39.1%)		0.006*
Action taken for rabid dog bite	Wash with soap	74(55.2%)	58(69.0%)	46(76.7%)	43(79.6%)	14(79.6%)	25.031	0.002*
	Healing with hot paper	40(29.9%)	22(26.2%)	13(21.7%)	9(16.7%)	4(17.4%)		
	Apply chili powder	20(14.9%)	4(4.8%)	1(1.7%)	2(3.7%)	5(21.7%)		
What action taken for affected person?	Bought medicine	40(30.3%)	29(34.9%)	22(36.7%)	38(76%)	14(56%)	12.531	0.014*
	Took the person to a traditional healer	37(28%)	21(25.3%)	20(33.3%)	14(28%)	4(16%)		
	Took the person to the nearest health facility	79(59.8%)	44(53.0%)	29(48.3%)	39(78%)	5(20%)		
	Did nothing	33(25%)	30(36.1%)	23(38.3%)	26(52%)	16(64%)		

Note that * indicates a significant difference at a *p* = 0.05, **X**^**2**^ = Chi-square

### Comparison of respondents’ perceptions regarding zoonosis with their residence

Overall, for comparison of respondents’ perceptions of zoonosis with their residence, 219 rural and 131 urban respondents were involved. The majority of rural participants reported outbreaks of anthrax (35.6%), tuberculosis (53.2%), and brucellosis (15.7%), while the outbreak of rabies was mentioned by urban residents (62.5%). The perceptions of respondents indicated that both rural (88.8%) and urban (89.6%) participants recognized that rabies can be transmitted through the bite of an infected dog, and most respondents understood that if not treated, this disease can cause behavioural change (70.6%), followed by death (65.9%), madness (67.7%), and not knowing (5.9%). The association between the transmission route and the fate of an unthreatened person for rabies with residence was not statistically significant (*p* > 0.05). Most respondents including urban (77.0%) and rural (70.6%) residents believed that the vaccination of dogs is important, and many reported that vaccination against brucellosis is available in their area (Table 5). The present study also revealed that nearly all respondents (uraban (98.5%), and rural (95.9%) reported that animal vaccination could prevent anthrax, and the majority of urban residents reported the availability of a vaccine against brucellosis in their area. The association of vaccination of dogs, availability of vaccine against brucellosis, and prevention of anthrax through vaccination with residence of respondents statistically not significant (*p* > 0.05).

**Table 5:** Perception of respondents on zoonosis in relation to residence(*n* = 350)

Variable	Responses	Residence Vs perception			
		Urban F (%) *n* = 131	Rural F (%) *n* = 219	X^2^	*P*- value
Is there outbreak of anthrax in your area?	Yes	41(30.1%)	77(35.6%)		
Does vaccination of animals help to prevent anthrax?	Yes	129(98.5%)	210(95.9%)	1.303	0.861
Do you have a dog?	Yes	77(58.8%)	113 (51.6%)	1.670	0.434
Is there the outbreak of the rabies in your area?	Yes	85(62.5%)	110(50.9%)		
How someone gets infected with rabies in your area?	Through saliva	79(58.5%)	92(42.8%)	0.166	0.920
	Bite of infected dog	120(89.6%)	191(88.8%)		
	Contact with rabid dog	67(50.0%)	88(40.9%)		
Fate of an untreated person bitten by a dog that has rabies	Behavioral change	96(70.6%)	164(75.6%)	2.097	0.350
	Death	86(63.2%)	143(65.9%)		
	Madness	90(67.7%)	109(50.7%)		
	Did nothing	8(5.9%)	11(5.1%)		
Is annual vaccination of dog against rabies necessary?	Yes	96(70.6%)	167(77.0%)	3.453	0.485
Is there the outbreak of tuberculosis in your area?	Yes	64(47.1%)	115(53.2%)		
Is there the outbreak of brucellosis in your area?	Yes	17(12.6%)	34(15.7%)		
Is vaccination available against brucellosis?	Yes	74(56.5%)	123(56.7%)	2.120	0.714

Note that * indicates a significant difference at a *p* = 0.05, **X**^**2**^ = Chi-square

### Some practice of participants with their monthly income

**Fig. 4:**
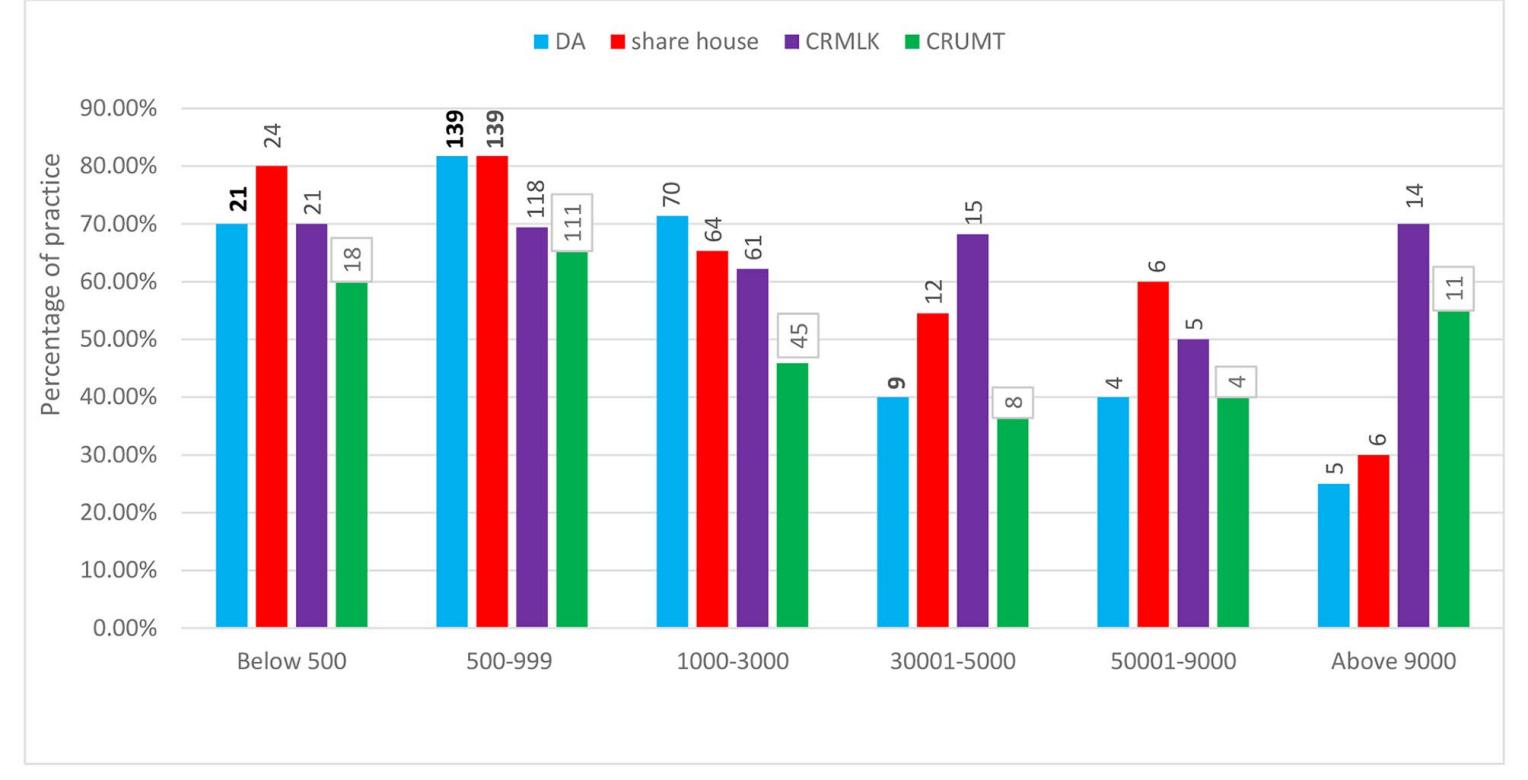
Monthly income Vs practice of respondents. Note: DA = respondents who have domestic animals, share house = respondents who share the same house with domestic animals, CRMLK = respondents who have the habit of consuming raw milk, CRUM = respondents who have the habit of consuming raw or undercooked meat

The majority of participants (81.8%) with a monthly income of 500–999 birr had domestic animals, and 81.8% of them shared the same house with their animals (Fig. 4). Among participants with a monthly income of 3001–5000/$50.02-$83.33, less than 500 birr/$8.33, 5001–9000 birr/$83.35-$150 and more than 9000/$150, 71.4%, 70%, 40% and 25% had domestic animals, respectively, and 54.5%, 80%, 60% and 30% of them, respectively, shared the same house with their domestic animals. More than half of the respondents with different monthly incomes reported consuming raw milk and undercooked or raw meat (Fig. 4).

## Discussion

The present study was conducted to assess the knowledge, perceptions, and practices of respondents regarding zoonotic disease and associated risk factors. The study indicated that the majority of respondents had high knowledge of bovine tuberculosis and rabies, but lower knowledge of brucellosis and anthrax was reported. Approximately 82.9%, 80%, 45.1%, and 24.3% of respondents had knowledge of bovine tuberculosis, rabies, anthrax, and brucellosis, respectively. These findings were lower than the findings of [21] in Addis Ababa, who reported that all respondents mentioned rabies (100%), followed by anthrax (94.3%), bovine tuberculosis (88.5%), and brucellosis (49.5%), and higher than the reports of [25] in and around Yabello districts, who reported bovine tuberculosis (19.8%), anthrax (15.6%) and brucellosis (14.8%). The possible reason for the variation in knowledge levels of respondents in different parts of the country could be the difference in access to information and education between urban and rural areas [10]. Urban areas such as Addis Ababa may have better access to health care facilities, veterinary services, and educational resources, leading to greater zoonotic knowledge than that of the inhabitants of rural or smaller cities such as the current study area [6].

A comparison of respondents’ knowledge with their sociodemographic factors indicated that male participants had greater knowledge of bovine tuberculosis (84.8%), rabies (79.8%), and anthrax (48.6%), while female respondents had greater knowledge of brucellosis (23.6%). This finding contrasts with a previous report of [26], stated that females had greater knowledge of anthrax (83.1%), rabies (97.1%), and tuberculosis (85.8%). The difference in knowledge levels between genders may be ascribed to variations in relationships with domestic animals and access to educational resources such as books, the internet, or media [26]. The lower knowledge of respondents on brucellosis was in agreement with previous studies [2, 21, 25–29], possibly due to a lack of responsiveness and a lack of well-trained veterinary and human health workers and very low cooperation among veterinary and human health office to implement one health strategy in developing countries [4, and 12].

A comparison of respondents’ knowledge with their education level and residence revealed that respondents with higher education levels (preparatory) had more knowledge of rabies (94%), bovine tuberculosis (84%), anthrax (48%), and brucellosis (24%), and urban residents had more knowledge of bovine tuberculosis (89.3%), rabies (83.2%), and anthrax (50.4%). Additionally, higher knowledge of brucellosis (24.2%) was reported by rural residents. This result was compared with the study of [30], who reported that more than 88% of college and university students and 50.4% of urban respondents had high knowledge of zoonotic anthrax, tuberculosis and rabies. Similarly [31], in Kenya stated that high school students had greater knowledge of zoonoses. The higher knowledge levels among students could be ascribed to their exposure to educational materials and curricula that cover these diseases.

Regarding the source of information, 68% of participants reported radio as their primary source of information, followed by TV (48.9%), family/friends (48%), teachers (44.3%), and veterinary health professionals (44%), and a low number of respondents reported newspaper and religious leaders as their source of information (Fig. 2). This finding is consistent with previous studies by [30] that identified electronic media such as radio and television as the major sources of information; in contrast [23] and [32], reported that 2.4% and 9% of respondents, respectively, reported veterinarians and animal health experts as their sources of information. This suggested that the decreased role of veterinarians and animal health professionals, coupled with the environmental expertise of the country, and it is important to set effective prevention and control measures through one health approach [12, and 15].

The majority of rural residents reported outbreaks of tuberculosis (53.2%), anthrax (35.6%), and brucellosis (15.7%), while 62.5% of urban residents reported rabies outbreaks in their area. The greater incidence of outbreaks of bovine tuberculosis, anthrax, and brucellosis in rural areas may be due to increased contact with domestic animals and shared living spaces [31]. Conversely, the higher rabies outbreak rates in urban areas are most likely due to a larger dog population (58.8%) [15]. These results are in agreement with the findings of [31], who reported that outbreaks of anthrax, brucellosis, tuberculosis and rabies in the South Omo Zone of the SNNP, Ethiopia [15], and a high incidence of rabies in three districts of Western Hararghe (Chiro, Hirna, and Mieso), Ethiopia [33], were reported as priority zoonoses in Côte d’Ivoire. More than half of the respondents mentioned the availability of vaccinations against brucellosis, with a significant proportion of urban (26.7%) and rural participants (48.8%) recognizing that brucellosis causes abortion in cattle, indicating a greater perception of brucellosis compared to the previous study of [29], who reported that only 1.3% of the respondents knew about vaccinating against brucellosis and 25.4% of respondents knew that brucellosis causes abortion in cattle and small ruminants; these findings are comparable to the findings of [34], who reported that 30% of the respondents had an incidence of abortion due to brucellosis in Kenya [34]. It is essential for government officials, NGOs, and community leaders to prioritize livestock and community health to effectively address these issues by strengthening one health approach to target human, animal and environmental health [4, and 12].

The study revealed that more than 52% of respondents had a high perception of the transmission of zoonotic disease from animals to humans, which is higher than that reported in previous studies [35] and [36], mentioned that 12.8% of respondents knew that diseases in animals can be transmitted to humans. Nearly all respondents agreed on the transmission of zoonoses through consuming raw milk and raw or undercooked meat, a practice that was prevalent in the study area. These findings are comparable to the results of [37], who reported that 96.5% and 66.2% of respondents consumed raw meat and milk, respectively, in the Lalo Kile District, Kellem Wollega Zone, Ethiopia. The majority of respondents (88.8%) recognized that rabies can be transmitted by the bite of infected dogs and that bovine tuberculosis can be transmitted from animal to human through the consumption of raw milk and meat. These findings are in agreement with reports [36] in Jimma, southwestern Ethiopia, who, reported that 94.3% of respondents believed that rabies can be transmitted through an infected dog bite and that tuberculosis can be transmitted from cattle to humans through inhalation and consumption of raw milk and meat. The differences in perception among respondents can be ascribed to a combination of factors such as education level, residence, availability of information sources, income, and cultural beliefs and practices [38].

In terms of respondents’ practices, the majority of respondents with primary (85.7%), secondary (80.0%), preparatory (85.2%), and diploma and above (56.5%) levels of education reported always washing their hands with soap after touching infected animals. This percentage was higher than the findings of previous studies [27] in Colombia (62.38%) [32], in the United States (45%) [39], in the Netherlands (50%) and [32] in Cheshire, England (15%), that reported washing their hands after contact with their pets. Regarding animal vaccination, 69.5% of respondents vaccinated their animals, and 70% recognized the importance of vaccinating dogs, which is comparable with previous studies in Addis Ababa, Ethiopia [27], reported that 65.3% of the respondents vaccinated their dogs and in contrasts with studies [37], reported that 25.6% of respondents vaccinated their dogs. Most respondents, regardless of educational level, stated that they would take an infected person to the nearest health center; this finding is consistent with the finding of [40] in Addis Ababa, where almost all respondents agreed to refer health professionals in case of animal bites. In contrast [40], , in and around Gondar town, Ethiopia, indicated that approximately 62.2% of the study participants had strong beliefs in traditional medicine. This difference may be due to variations in educational levels, respondents’ perceptions of traditional medicine and health care, income, and cultural beliefs and practices in different localities [38]. These cultural practices may be deeply rooted in traditions and may require targeted interventions to change behaviours and improve knowledge and practices [13, 16].

In terms of income, the majority of participants (81.8%) with a monthly income of 500–999 birr/$8.33–$16.65 reported having domestic animals and sharing the same house with them. These results are higher than the previous findings of [41] in Ethiopia, who reported that 40.4% of respondents shared the same house with animals. Participants with different income levels practiced consuming raw milk and raw meat (Fig. 3). In contrast [42], , in Kars, Turkey, reported that monthly income did not affect knowledge or attitudes; however, positive practices were more common among farmers with higher income levels who had better access to information and resources, allowing them to take proper precautions [42]. Addressing these factors through targeted interventions, the implementation of one health approach, education campaigns, and improved access to information and resources can help bridge the knowledge gap and improve practices related to zoonotic diseases. In addition, including zoonoses in the curriculum from lower levels of education and implementing zoonotic programs on media platforms can help raise awareness and improve perception [38, and 43]. Some studies have shown that local people sometimes prefer traditional medicine for their livestock disease due to their cultural and spiritual perspectives, proven effectiveness, easy access and low financial cost of sourcing [44–46].

Knowledge, perception, and practice studies are important for evaluating the presence of knowledge in the community and documenting respondents’ practices that are likely to increase the risk of zoonoses. Findings from these studies are crucial for providing baseline information and guiding public health education programs that attempt to fill existing knowledge gaps and reduce the practices of communities that potentially favour pathogen transmission. The limitations of this study were that only a few zoonotic diseases that were purposively selected were assessed, which could affect the generalization of the whole zoonosis, and the role of wild life in the transmission of zoonosis is also undermined. This study also focused on a few risks of zoonosis, and the importance of one health was not assessed as the main target of the study, although the role of one health was appreciated in controlling zoonoses.

## Conclusion

Generally, higher knowledge of bovine tuberculosis and rabies was reported, and in contrast, lower knowledge of anthrax and brucellosis was reported by the researcher in the present study. Overall, although the reported knowledge and perceptions of respondents are promising, more improvements are needed. Considering the association of respondent knowledge, perception, and practice with different sociodemographic factors, a correlation was found with education level, residence, and income. A higher education level was associated with better hand washing practices, animal vaccination, and knowledge of appropriate actions in the case of an animal bite. However, it is important to note that there may be other factors, such as cultural beliefs and access to healthcare services may influence these practices. Further research is needed to better understand these factors and develop targeted interventions that involve the targeted implementation of one’s health approach to improve the practices of community and to spread zoonotic-related information in a full manner.

### Electronic supplementary material

Below is the link to the electronic supplementary material.

Supplementary Material 1

## Data Availability

The data used in the studies are available from the corresponding author upon reasonable request.

## Abbreviations

CSA: Central Statistical Agency
FAO: Food and Agriculture Organization of the United Nations
NOHS: National One Health Strategy
OIE: World Organization for Animal Health
SPSS: Statistical Package for Social Science
WHO: World Health Organization
