# Supplementary material for: Associations of community knowledge, perceptions, and practices related to zoonotic disease with sociodemographic factors in and around Chiro Town, Eastern Ethiopia: a cross-sectional study
Source: One Health Outlook. 2024 Jun 7;6:10. doi: 10.1186/s42522-024-00105-9 (PMC11157839; doi:10.1186/s42522-024-00105-9)
Supplement: Supplementary file 1 — Supplementary Material 1 [file 42522_2024_105_MOESM1_ESM.docx]

**ODA BULTUM UNIVERSITY**

**COLLEGE OF NATURAL AND COMPUTATIONAL SCIENCES**

**Questioners** **on** **Association of community knowledge, perception, and practice related to zoonotic disease with sociodemographic factors in and around Chiro Town Eastern Ethiopia: A cross-sectional study**

**Dear respondent!**

This questionnaire was designed to collect a data for a research entitled with “**Association of community knowledge, perception, and practice related to zoonotic disease with sociodemographic factors in and around Chiro Town Eastern Ethiopia: A cross-sectional study**” from Oda Bultum University. This survey questionnaire was designed with the objective of assessing the conception and practices of the public about zoonotic diseases in West Hararghe zone. For this purpose your genuine responses to each of the survey questions are highly useful. Your answers will be confidential and needed only for investigation. You may, at any time, with draw your participation, including the withdrawal of any information you have provided. We highly appreciate for your willingness to participate as a respondent in this survey.

**Preliminary Information**

1. Questionnaire Code __________________
2. Interviewer’s name _____________________ Tell___________________________
3. District ___________ Kebele ______Village __________
4. Sex
5. Male ______
6. Female ________
7. Marital Status
8. Married_______
9. Unmarried ______
10. Divorced_______
11. Educational Status
12. No formal education ____
13. Primary education _____
14. High school____
15. Preparatory school ____
16. Diploma and above ____
17. Residence/ Location

A. Urban _____ B. Rural _____

1. Occupation

A. Teacher ____ B. Student ____ C. Farmer ____ D. other ______

1. Family Size

A.1_____ B.2-5 ______ C. 6 and above ______

1. Monthly Income

A. 500 - 999 _____ B. 1000 - 3000 ______ C. 3001-5000____ D. 5001-8000 ______ E. 9000 and above _______

8. Do you have any domestic animals?

A. Yes B. No

**II. Questions to evaluate the Knowledge of the participants**

1. Which of the following diseases transmit from animals to human being

A. Rabies

B. Bovine tuberculosis

C. Anthrax

D. Brucellosis

1. From the following which disease do you know and mention its sign

| Do you know | Yes | No | Sign |
| --- | --- | --- | --- |
| Rabies |  |  |  |
| TB |  |  |  |
| Anthrax |  |  |  |
| Brucellosis |  |  |  |

1. How does humans contract with zoonotic disease?

| **How Does Human Get Infected By Zoonotic Diseases?** | **Agree** | **Disagree** |
| --- | --- | --- |
| Eating raw or under cooked meat |  |  |
| Drinking raw milk |  |  |
| Bite from infected animal |  |  |
| Inhalation |  |  |
| Handling animals with cut or wounds |  |  |
| Through vectors |  |  |
| Through food |  |  |
| Sharing the same room with animals can expose to zoonoses |  |  |
| Drinking of water contaminated with infected animal feces |  |  |
| Ingestion of contaminated soil |  |  |

1. Which of the following is important source of the transmission of zoonotic disease?

A. dog B. cat C. poultry D. cattle/buffalo E. sheep/goat

1. Do you know that tuberculosis can be transmitted from cattle to humans?

A. Yes B. No

14. Where did you learn about zoonotic disease? (check all mentioned)

A. Newspapers and magazines [ ]

B. Radio [ ]

C. TV [ ]

D. Veterinary officials [ ]

E. Brochures, posters and books [ ]

F. Teachers [ ]

G. Religious leaders [ ]

H. Family, friends, neighbors/colleagues [ ]

I. Other (Specify) …………..……………...

15. What do you think causes zoonotic disease?

A. Germs

B. Hereditary

C. Witchcraft

D. Don’t know

16. How is zoonotic disease transmitted to humans? (Select all that apply)

A. Eating infected animal product [ ]

B. By handling infected animals without protective clothing [ ]

C. Through contaminated soil [ ]

D. Do not know [ ]

17. Have you ever seen a person with rabies, anthrax, brucellosis?

A. Yes [ ] B. No [ ]

18. If yes, where?

A. Through media (TV) [ ] B. Personal observation [ ] C. Other (specify) ……...………

19. How can a person prevent him/herself from getting rabies, anthrax, and brucellosis ? (Select all that apply)

A. Avoid infected animals with those disease [ ]

B. Avoid infected people with those disease [ ]

C. Burn all suspected animal carcasses 4-Bury all suspected carcasses [ ]

D. Vaccinate animals annually [ ]

E. Do not know [ ]

20. How someone get infected with rabies?

A. Animal bite

B. Contact with rabid dog

C. Through saliva of infected dog

**III. Questions to assess the perception of respondents towards zoonotic disease**

1. What do you think about the fate of an untreated person bitten by a rabid dog?
2. death
3. madness
4. behavioral change
5. development of puppies in the abdominal cavity of the person
6. I do not know what would happen to the person.
7. Which of the following could be risk of raw meat and milk consumption

A. Diarrhea B. tuberculosis C. typhoid D. amoeba

1. Do you have habit of consuming raw meat?

A. Yes B. No

24. If your answer for the above question is yes, don’t you worry about your health?

A. Not worried B. Worry

1. If you see a dead animal nearby your home, what do you feel?
2. Not concerned
3. Somewhat concerned about bad smell
4. Very concerned about possible diseases
5. Answer the following question accordingly

| s/no | Question |  |  |
| --- | --- | --- | --- |
| 1 | Have you ever got disease transmitted to you from your/others animal | Yes | No |
| 2 | Do you know Brucellosis can cause abortion in dairy animals? | I know | I don’t know |
| 3 | Do we need vaccination after rabid dog bite? | Yes | No |
| 4 | Is vaccination available against brucellosis? | Yes | No |
| 5 | What shall we do for rabid dog bite wound? | Wash with soap | Apply chili powder |
| 6 | Is annual vaccination of dog against rabies is necessary? | Yes | No |

1. Identify the possible means of contracting zoonoses from the various types of animal products

| S/No | Animal products | Contracting zoonoses | |
| --- | --- | --- | --- |
| 1 | Cows’ milk | Yes | No |
| 2 | Goats’ milk | Yes | No |
| 3 | Beef | Yes | No |
| 4 | Goat meat | Yes | No |
| 5 | Mutton | Yes | No |
| 6 | Chicken meat | Yes | No |
| 7 | Chicken eggs | Yes | No |

**III. Questions to assess the practice of the respondents**

1. If you see a dead animal nearby your home, what do you feel?
2. Buried it or burn it
3. Throw it away on the field
4. Do nothing
5. Do you wash your hands with soap after touching animals to prevent zoonotic diseases?
6. Yes I do it always
7. No I don‘t
8. Yes I do it some times
9. I wash only when I eat food
10. Do domestic animals in your community share house with people?
11. Yes they do.
12. No, they don‘t at all.
13. Yes, they do in some few cases
14. If your answer to question number 30 is A or C do you worry about the health of these people?
15. Yes I do
16. There is no reason to worry
17. When cattle die of disease, how do you handle?
18. Throw it away on the field
19. Use it for pets as food
20. Buried it or burn it
21. Give answer for the following questions

|  |  | Yes | No |
| --- | --- | --- | --- |
| 1. | If you have a dog do you vaccinate your dogs regularly? |  |  |
| 2. | Do you allow your dog to roam outside your compound? |  |  |
| 3. | Do you consume raw meat? |  |  |
| 4. | Have you ever encountered a rabid dog? |  |  |

1. Have you ever noticed rabies, Anthrax, Brucellosis in your domestic animals?

A. Yes B. No C. I don’t know

1. If your answer for the above question is yes, what actions did you take?

A. Reported to the Vet officer [ ] B. Buried the dead animal without reporting [ ]

C. Consumed meat of the dead animal [ ] D- Other (Specify) …………………

1. Has any member of your family suffered from Rabies, anthrax, TB, Brucellosis?

A. Yes [ ] B. No [ ] C. Don’t know [ ]

1. If your answer for the above question was yes above, how did the person contract it?

A. Skinning dead animal [ ] C. Other (Specify)……………..

B. Eating dead animal [ ]

1. If a member of your family is infected with rabies, brucellosis, anthrax or TB; what action did you take?

A. Took the person to the nearest health facility [ ]

B. Bought medicine from a chemist (Specify drugs bought) ………….…………

C. Took the person to a traditional healer [ ]

D. Did nothing [ ]

E. Other (Specify) ……………………………………….

1. Have you had an outbreak of the following zoonotic disease in your area? Yes [ ] No [ ]

A. Rabies C. Anthrax

B. Tuberculosis D. Brucellosis

1. If yes, which one and when?

A. Less than 6 months ago [ ] B. More than 1year ago [ ]

C. Don’t remember [ ]

1. Was there vaccination during the period? Yes [ ] No[ ]
2. Were all animals vaccinated? (tick)- Yes [ ] No [ ]
3. If No which animals were not vaccinated and why?-------------------------------------------------
4. How often is vaccination against anthrax done in your area?
5. Twice a year [ ]
6. Once a year [ ]
7. The veterinary personnel are always available to vaccinate
8. Never available [ ]
9. Other (specify)..............................................
10. What prompts you to take your animals for vaccination?
11. To protect animals [ ] B. Because others do so [ ]
12. To protect humans [ ] D. Because it is a requirement [ ]
13. Other (Specify)..................................
14. If you do not always take your animals for vaccination, what are the reasons?
15. No Vet services [ ]
16. The vaccination center is far [ ]
17. Financial difficulties [ ]
18. Don’t get informed when it occurs [ ]
19. Other (Specify)................................
20. In your opinion, does vaccination of animals help to prevent anthrax?
21. Yes [ ] B. No [ ] C. why? …………………………………
